# New Insight into Isoprenoids Biosynthesis Process and Future Prospects for Drug Designing in *Plasmodium*

**DOI:** 10.3389/fmicb.2016.01421

**Published:** 2016-09-13

**Authors:** Gagandeep S. Saggu, Zarna R. Pala, Shilpi Garg, Vishal Saxena

**Affiliations:** Molecular Parasitology and Systems Biology Laboratory, Department of Biological Sciences, Birla Institute of Technology and SciencePilani, India

**Keywords:** *Plasmodium*, apicoplast, isoprenoids, MEP pathway, anti-malarial

## Abstract

The MEP (Methyl Erythritol Phosphate) isoprenoids biosynthesis pathway is an attractive drug target to combat malaria, due to its uniqueness and indispensability for the parasite. It is functional in the apicoplast of *Plasmodium* and its products get transported to the cytoplasm, where they participate in glycoprotein synthesis, electron transport chain, tRNA modification and several other biological processes. Several compounds have been tested against the enzymes involved in this pathway and amongst them Fosmidomycin, targeted against IspC (DXP reductoisomerase) enzyme and MMV008138 targeted against IspD enzyme have shown good anti-malarial activity in parasite cultures. Fosmidomycin is now-a-days prescribed clinically, however, less absorption, shorter half-life, and toxicity at higher doses, limits its use as an anti-malarial. The potential of other enzymes of the pathway as candidate drug targets has also been determined. This review details the various drug molecules tested against these targets with special emphasis to *Plasmodium*. We corroborate that MEP pathway functional within the apicoplast of *Plasmodium* is a major drug target, especially during erythrocytic stages. However, the major bottlenecks, bioavailability and toxicity of the new molecules needs to be addressed, before considering any new molecule as a potent antimalarial.

## Introduction

Isoprenoids are structurally and functionally the most diverse group of natural metabolites found in all three domains i.e., eubacteria, archaebacteria and eukarya. Depending on the number of precursor units [Isopentenyl Pyrophosphate (IPP) and Dimethylallyl Pyrophosphate (DMAPP)], they vary in structure and functions. Isoprenoids are known to play a key role in all aspects of life; e.g., in regulation of gene expression (prenylation of proteins), as membrane constituents (prenyl lipids in archaebacteria and sterol in eubacteria and eukaryotes), as vitamins, plant hormones (gibberellins, brassinosteroids, abscisic acid), photosynthetic pigments (carotenoids, side chain of chlorophyll), quinones in electron transport chain, and plant defense compounds (monoterpenes, sesquiterpenes, diterpenes) (Sacchettini and Poulter, 1997; Bach et al., 1999; Hunter, 2007). Two distinct pathways synthesize IPP and DMAPP, (a) the Mevalonate dependent pathway (MVA) functional in archaea and most eukaryotes (including all mammals and higher plants), and (b) the Methyl Erythritol Phosphate/1-deoxy-D-xylulose-5-phosphate (MEP/DOXP) pathway functional in bacteria, plant plastids, members of chlorophyta and pathogenic microorganisms (Banerjee and Sharkey, 2014). These pathways are significantly different from each other (Figure S1) in terms of preliminary substrates, mevalonate as an intermediate in MVA pathway only, and higher yield of IPP and DMAPP in MEP pathway.

### Isoprenoids biosynthesis in *Plasmodium*

*Plasmodium* belongs to the phylum Apicomplexa, known to harbor a non-photosynthetic plastid like organelle of prokaryotic origin known as apicoplast (McFadden et al., 1996; Foth and McFadden, 2003). This organelle is indispensable for the survival of the parasite and is the functional site for four major metabolic pathways. The MEP/DOXP pathway is one of these pathways which is the only source for isoprenoids in the parasite and is absent in the human host. The first evidence for the presence of the MEP pathway in *Plasmodium* was given by Jomaa et al. (1999) who identified the presence of DOXP reductoisomerase (IspC) gene in preliminary staged *Plasmodium falciparum* whole genome database. Following this, few other enzymes of this pathway, IspD (Rohdich et al., 1999), IspF (Rohdich et al., 2001), IspG (Altincicek et al., 2001a), and IspH (Altincicek et al., 2001b) were characterized mainly from prokaryotes and were shown to be present in *P. falciparum* as well. Studies have detailed the import of initial substrates of the pathway, DHAP (Dihydroxy acetone phosphate) and PEP (Phosphoenol pyruvate) inside the *Plasmodium* apicoplast with the help of transporter molecules TPT (triose phosphate transporter) and PPT (phosphoenol pyruvate transporter) respectively, localized in the apicoplast membrane (Mullin et al., 2006) suggesting apicoplast as its functional site.

In *Plasmodium*, the MEP pathway is reported to be indispensable for both hepatic (Sparr et al., 2013) and erythrocytic stages (Cassera et al., 2004) of the parasite asexual life cycle. Recent reports have suggested that the “apicoplast less” *Plasmodium* parasite can divide indefinitely in culture if supplemented exogenously with IPP. This proves that during the erythrocytic stages, the only essential function of apicoplast is the synthesis of isoprene unit precursors, IPP and DMAPP (Yeh and DeRisi, 2011). Recent reports have also proven that the products of MEP pathway are required in the early stages of parasite gamete development (Wiley et al., 2015). All these studies suggest the importance of this pathway at different phases of the parasite's life cycle.

### MEP pathway enzymes

The MEP pathway consists of seven enzymes, encoded by the parasite nuclear genome and targeted to apicoplast with the help of N-terminal bipartite leader sequence (van Dooren et al., 2002). While all these enzymes are well characterized in prokaryotes like *Escherichia coli*, only a few have been detailed from pathogenic organisms including *Plasmodium*. Here, we have detailed a comparative analysis of each enzyme of the pathway from different organisms and discussed the various natural and chemically derived molecules used as their inhibitors in recent years with special emphasis to *Plasmodium* (Table 1).

**Table 1:** **Inhibitors reported for different enzyme involved in the MEP pathway with their chemical properties**.

**Gene/Protein**	**Enzyme**	**Inhibitor molecules**		**Inhibition mechanism**	**Organism with IC_50_ value**	**References**
DXS	1-deoxy-D-xylose-5-phosphate (DXP) Synthase	^*^6-benzyl-3-(4-chlorophenyl)-5-methyl-2-(trifluoromethyl)pyrazolo[1,5-a]pyrimidin-7(4H)-one	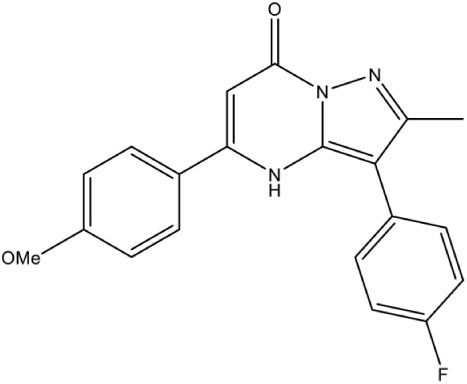	Competitive inhibition	*M. tuberculosis* (IC_50_10.6 μM)	Mao et al., 2008
		Ketoclomazone PubChem CID: 12811046	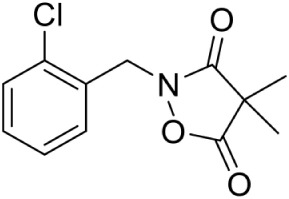	Non competitive inhibition	*E. coli* (IC_50_800 μg/mL) and *H. influenzae* (IC_50_12.5 μg/mL)	Matsue et al., 2010
		β-fluoropyruvate PubChem CID: 67946	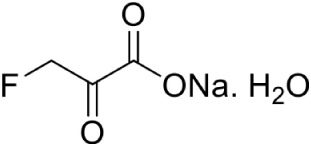	Competitive inhibition	*P. vivax* (IC_50_35 ± 1.7 μM) *P. falciparum* (IC_50_43 ± 3.8 μM)	Battistini et al., 2016
		Methylacetylphosphonate PubChem CID: 23674726	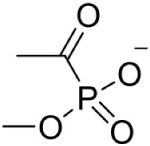	Competitive inhibition	*P. vivax* (IC_50_80 μM) *P. falciparum* (IC_50_46 ± 3.8 μM)	
IspC	DXP reductoisomerase	Fosmidomycin PubChem CID: 572	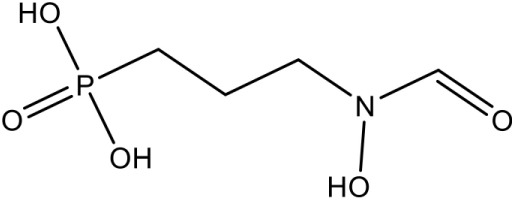	Competitive inhibition	*P. falciparum* (IC_50_350 ± 170 nM)	Jomaa et al., 1999; Lell et al., 2003; Umeda et al., 2011
		FR900098 PubChem CID: 162204	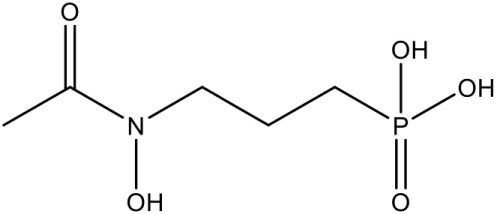	Competitive inhibition	*P. falciparum* (IC_50_170 ± 100 nM)	Jomaa et al., 1999
		^*^[1-(3,4-Difluorophenyl)-4-(hydroxylamino)-4-oxobutyl] phosphonic acid (Fosmidomycin reverse derivative)	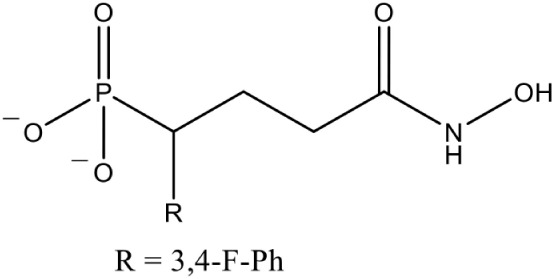	Competitive inhibition	*P. falciparum* (IC_50_3 nM)	Behrendt et al., 2011
		^*^((3,4-Difluorophenyl)(2-(hydroxy(methyl)amino)-2-oxoethoxy)methyl) phosphonic acid	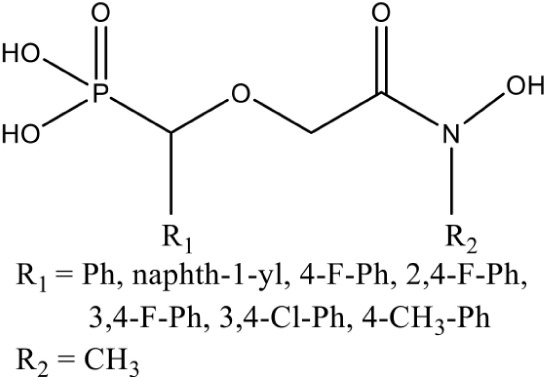	Competitive inhibition	*P. falciparum* (IC_50_12 nM)	Brücher et al., 2012
		^*^4-[Hydroxy(methyl)amino]-1-(4-methoxyphenyl)-4-oxobutylphosphonic acid (Fosmidomycin reverse analog	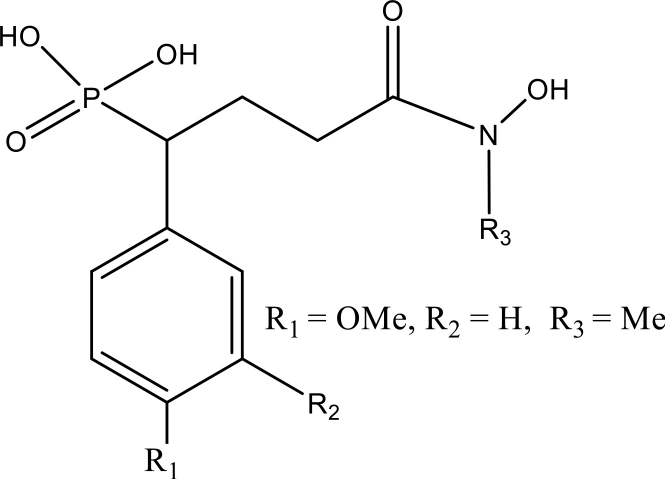	Competitive inhibition	*P. falciparum* (IC_50_20 nM)	Konzuch et al., 2014
IspD	2-C-methyl-D-erythritol 4-phosphate cytidylyltransferase	^*^L-erythritol-4-phosphate	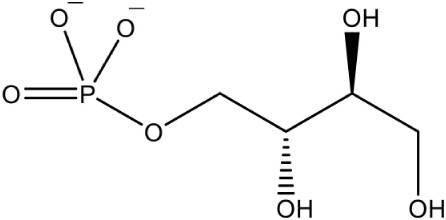	Competitive inhibition	*B. abortus* (IC_50_1.36 mM)	Lillo et al., 2003
		7-hydroxy-[1,2,4] triazolo [1,5-a] pyrimidine PubChem CID: 75629 (2503-56-2)	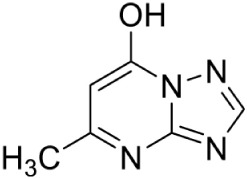	Allosteric inhibition	*A. thaliana* (IC_50_140 ± 10 nM)	Witschel et al., 2011
		^*^6-Amino-7-(1*H*-benzo[*d*]imidazol-2-yl)-5-[5-(diethylamino)-1-methylbut-1-yl)-5*H*-pyrrolo [3,2-*b*] pyrazine-2,3-dicarbonitrile	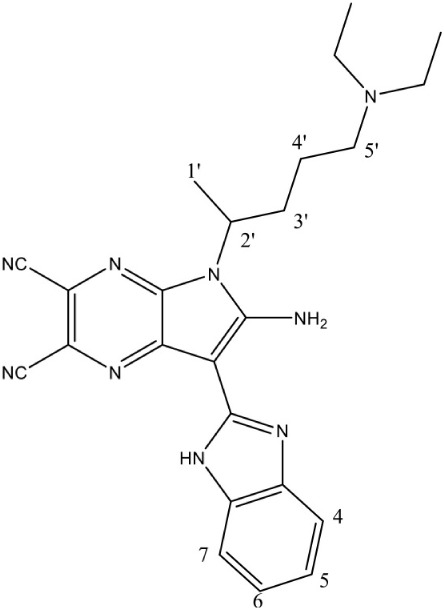	Non competitive inhibition	*P. falciparum* (EC_50_ 50 nM)	Reker et al., 2014
		Pyrroloquinoxaline	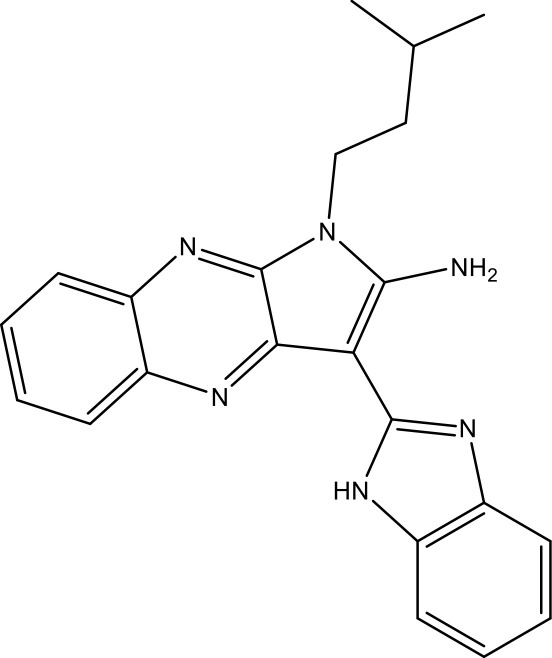	Non competitive inhibition	*A. thaliana* (IC_50_ 1.6 μM)	Reker et al., 2014
		MMV008138 PubChem CID: 2829106	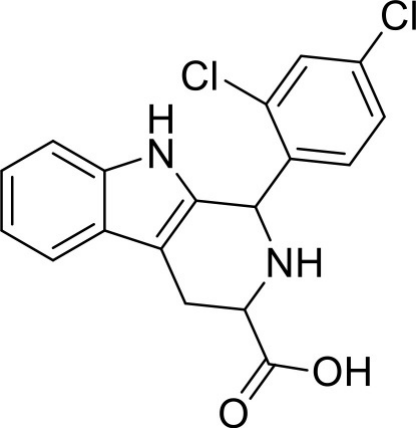	Competitive inhibition	*P. falciparum* (IC_50_47 nM) and *P. vivax* (IC_50_310 nM)	Imlay et al., 2015
		^*^Ethyl {3-[4-amino-5-{3-[(cyclopropylsulfonyl) amino] prop-1-yn-1-yl}-2-oxopyrimidin-1(2H)-yl] oxetan-3-yl} acetate	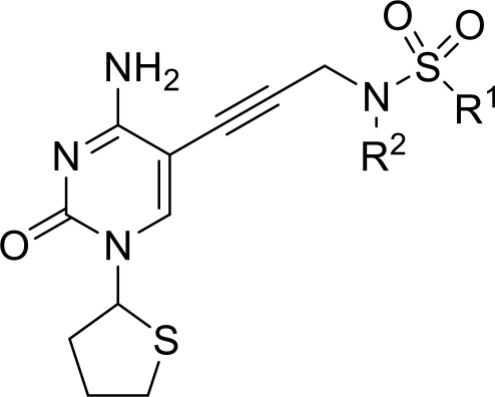	Competitive inhibition	*A. aeolicus* (IC_50_590 ± 10 nM)	Hirsch et al., 2008
IspE	4-(cytidine-5-diphospho)-2-C-methyl-D-erythritol kinase	6-(benzylthio)-2-(2-hydroxyphenyl)-4-oxo-3,4-dihydro-*2H*-1,3-thiazine-5-carbonitrile PubChem CID: 3768522	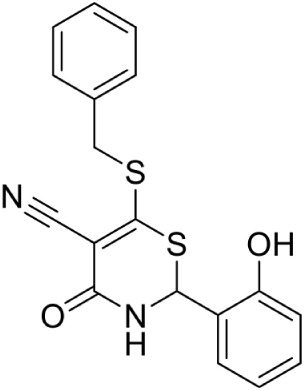	Competitive inhibition	*E. coli* (IC_50_5.5 μM)	Tang et al., 2011
		^*^Diammonium 5′-O-{[({[2-({[5-(Dimethylamino) naphthalene-1- yl]sulfonyl}amino) ethyl] oxy}phosphinato)oxy] phosphinato} cytidine	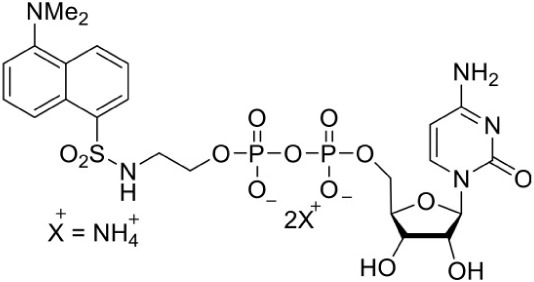	Competitive inhibition	*E. coli* (IC_50_3.0 μM)	Crane et al., 2006
IspF	2C-Methyl-D-erythritol-2, 4-cyclodiphosphate synthase	Thiazolopyrimidine PubChem CID: 330031	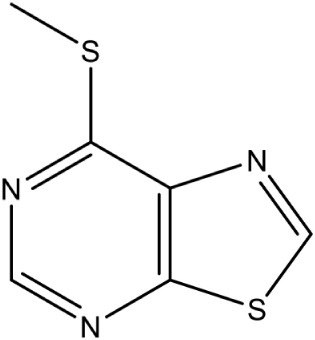	Competitive inhibition	*P. falciparum* (IC_50_9.6 μM) and *M. tuberculosis* (IC_50_6.1 μM)	Geist et al., 2010
		Aryl bis sulphonamide PubChem CID: 5333	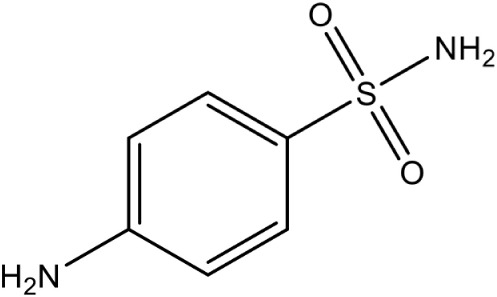	Competitive inhibition	*P. falciparum* (IC_50_1.4 μM) and *A. thaliana* (IC_50_240 nM)	Thelemann et al., 2015
		Propargyl diphosphate PubChem CID: 46236597	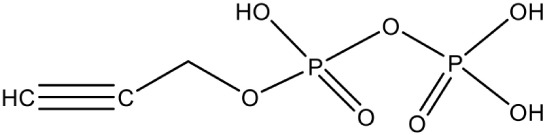	Competitive inhibition	*E. coli* (IC_50_750 nM)	Wang et al., 2010
IspG	4-Hydroxy-3-methyl-2-(E)-butenyl-4-diphosphate synthase	Prop-2-yn-1-yl trihydrogen diphosphate PubChem CID: 448670	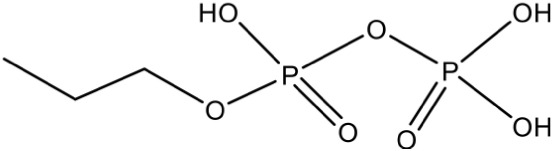	Competitive inhibition	*T. thermophilus* (IC_50_770 nM)	Quitterer et al., 2015
		But-3-yn-1-yl trihydrogen diphosphate PubChem CID: 46236598	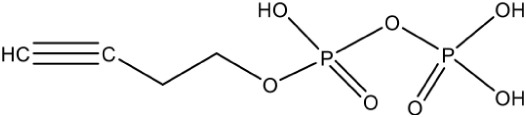	Competitive inhibition	*T. thermophilus* (IC_50_580 nM)	Wang et al., 2010
		But-3-yn-1-yl trihydrogen diphosphate PubChem CID: 46236598	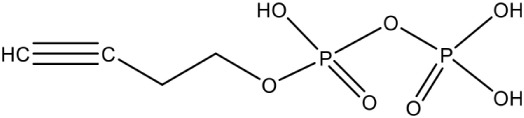		*A. aeolicus* (IC_50_450 nM)	
IspH	4-Hydroxy-3-methyl-2-(E)-butenyl-4-diphosphate reductase	Pyridine phosphate PubChem CID: 10866885	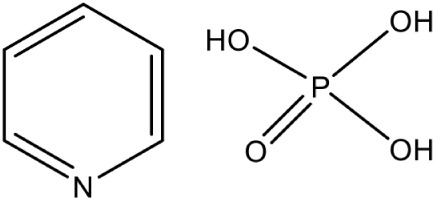	Competitive inhibition	*A. aeolicus* (IC_50_35 μM)	Wang et al., 2011
		^*^(E)-4-mercapto-3-methyl but-2 enyl diphosphate (Alkyne diphosphate derivative)	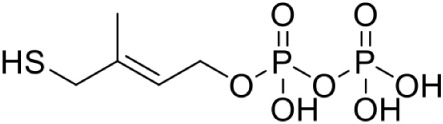	Competitive inhibition	*E. coli* (IC_50_210 nM)	Janthawornpong et al., 2013

* *PubChem Id not available*.

#### DXS (DXP synthase)

The initial substrates pyruvate and Glyceraldehyde-3-Phosphate are acted upon by a thiamine pyrophosphate dependent enzyme DXS (EC 4.1.3.37) to generate 1-deoxy-D-xylose-5-phosphate (DOXP/DXP) (Sprenger et al., 1997). This first step is believed to be rate limiting in some organisms and represents a branch point for Vitamin B and isoprenoids synthesis in bacteria (Hahn et al., 2001). The X-ray crystal structure of DXS enzyme has been elucidated from *E. coli* (PDB Id: 2O1S) and *Deinococcus radiodurans* (PDB Id: 2O1X). This enzyme consists of three functional domains: Thiamine Pyrophosphate (TPP/ThDP) binding domain, Pyrimidine (PYR) binding domain and transketolase C domain. In *E. coli*, the interaction of ThDP and Mg^2+^ has been observed with amino acid residues present in the TPP DXS domain (Xiang et al., 2007). However, in *Plasmodium*, the characterization of DXS from both *P. falciparum* and *P. vivax* suggests its existence as a homodimer (Handa et al., 2013) which contains one-bound Mg (II) per enzyme molecule, having both a catalytic and structural role in the enzyme. All ThDP dependent enzymes catalyze two successive half reactions. The first step involves the attack of an activated ThDP ylide on the first substrate GA3P. The next step can occur *via* three distinct mechanisms: (i) the most common classical ping-pong mechanism; (ii) through an ordered sequential kinetic mechanism or (iii) through an alternate random sequential mechanism (Brammer et al., 2011). Enzyme kinetics of DXS in *Plasmodium* points toward a random sequential kinetic mechanism, an unusual finding for ThDP-dependent enzymes similar to *Rhodobacter capsulatus* (Battistini et al., 2016).

In *Mycobacterium tuberculosis*, transketolase inhibitors like derivatives of Pyrimidinone (IC_50_ = 10.6 μM) have been shown to inhibit DXS enzyme (Mao et al., 2008). Later, a herbicide Ketoclomazone was shown to inhibit the DXS enzyme in both *E. coli* and *Haemophilus influenzae* by binding to a site, which differs from both the pyruvate and GA3P binding sites, thus suggesting non-competitive inhibition (Matsue et al., 2010). However, yet, the binding site of the drug is not elucidated. In another study, Smith et al. (2014) has demonstrated the selective inhibition of ThDP dependent enzymes in *E. coli, Yersinia pestis*, and *M. tuberculosis* by unnatural by-substrate analog butylacetylphosphonate (BAP) and its synergistic action with established anti-microbial agents like Fosmidomycin (Fos). However, BAP exhibits weak anti-microbial activity, possibly due to poor cellular uptake. Recently, analogs of pyruvate, β-Fluoropyruvate (F-Pyr) and methylacetylphosphonate (MAP) were demonstrated as competitive inhibitors of the DXS enzyme in both *P. falciparum* and *P. vivax* (Handa et al., 2013; Battistini et al., 2016).

#### IspC (DXP reductoisomerase/DXR)

IspC (EC 1.1.1.267) is the first enzyme committed to the isoprenoids biosynthesis in the MEP pathway. In a rate limiting step, it is responsible for the conversion of DOXP to 2-C-methyl-D-erythritol-4-phosphate (MEP) by using the NADPH *pro-S* hydride (Brammer et al., 2011). Its structure has been well defined in *E. coli* (PDB Id: 1Q0L; Mac Sweeney et al., 2005), *P. falciparum* (PDB Id: 3AU9; Umeda et al., 2011, 2015) and various other pathogens, including *M. tuberculosis* (PDB Id: 2Y1C; Andaloussi et al., 2011) and *Zymomonas mobilis* (PDB Id: 1ROK; Ricagno et al., 2004; Henriksson et al., 2006). It is a class B dehydrogenase enzyme that exists as a homodimer where each subunit is composed of three domains; an N-terminal domain for cofactor binding (DXP_reductoisom), central domain having active site residues (DXP_redisom_C) and a C-terminal helical domain (DXPR_C). The N-terminal domain is a member of dinucleotide binding fold and serves for binding of NADPH. The central catalytic domain harbors the binding sites for divalent cations (like Mn^2+^ or Mg^2+^), phosphate of the substrate and the catalytic loop. The C-terminal domain is connected to the catalytic domain by a linker region that spans entire monomer and appears to have a structural role in supporting the catalytic domain. Normally, the active site has two different conformations, open and closed. The open conformation allows the substrate, DXP, to enter and bind to the active site while in the closed conformation, a flap covers the active site and catalytic function is activated (Mac Sweeney et al., 2005). In *P. falciparum* IspC, NADPH molecule bind to the cavity composed of conserved residues D231, E233, S269, S270, W296, M298, S306, N311, K312, and E315 present toward the C-terminal of the enzyme. These residues are conserved in all human malaria parasites (Kunfermann et al., 2013).

**Figure 1:**
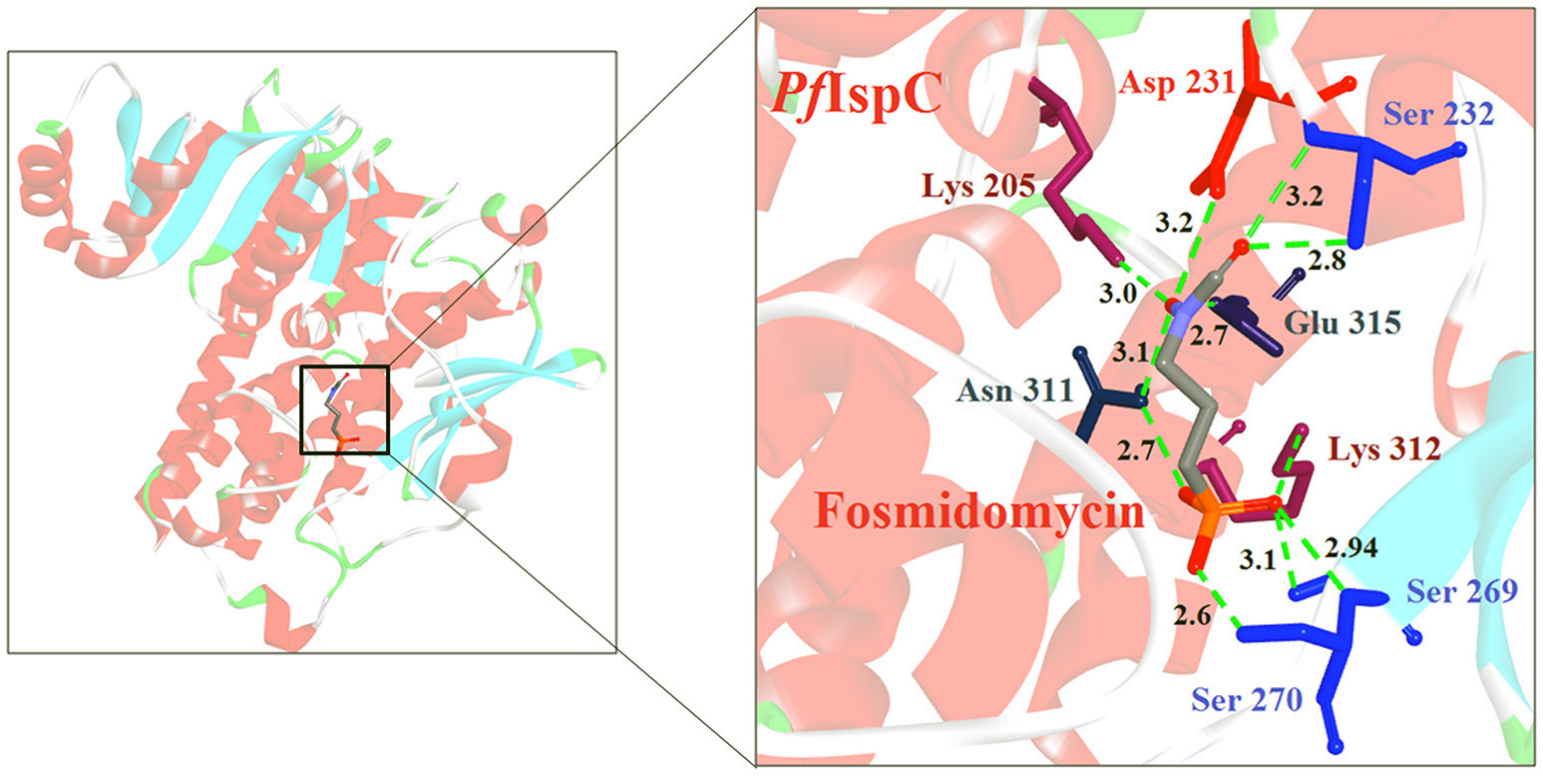
**X-ray crystal structure (PDB Id: 3AU9; Umeda et al., 2011) of *P. falciparum* IspC enzyme interacting with Fosmidomycin (represented as stick)**. The drug interacts with K205, D231, S232, S269, S270, N311, K312, and E315 residues of enzyme. CPK color scheme followed and distance represented in Å.

In *E. coli* and *P. falciparum*, Fos has been characterized as an inhibitor (IC_50_ = 0.032 μM) of IspC enzyme. It behaves as a substrate analog of DOXP and competes for its binding site on IspC (Jomaa et al., 1999; Steinbacher et al., 2003). In *P. falciparum*, Fos interacts with K205, D231, S232, S269, S270, N311, K312, and E315 residues of the catalytic domain (Figure 1; Umeda et al., 2011). The effect of Fos has been reported to be varied amongst different stages of *Plasmodium* development as well as amongst different apicomplexans. It was found that Fos is only effective on the erythrocytic stages of *Plasmodium* due to the formation of new permeability pathways (minute anionic selective channels formed due to entry of parasite in erythrocytes), but has no or minimal effect on the liver stages of *P. berghei*. In addition, Fos is not effective on many apicomplexans including *Toxoplasma, Eimeria* even at higher concentrations, probably due to its impermeability to the parasite plasma membrane (Baumeister et al., 2011; Nair et al., 2011).

Fos is one of those few drugs which was able to qualify the Phase–II clinical trials (in combination with Clindamycin), however, its treatment faces several drawbacks, including low bioavailability, rapid clearance from the parasite, less absorption (16.6 μg/mL after an oral dose of 40 mg/kg) and shorter half–life (1.14 h) leading to incidences of recurrence. Due to its toxicity at higher doses, this drug alone could not act as potential antimalarial (Murakawa et al., 1982; Kuemmerle et al., 1987; Lell et al., 2003). To overcome the bottlenecks in the use of Fos as an anti-malarial, different groups have synthesized analogs of Fos. Jansson et al. (2013) reported various analogs of Fos but only one among these, a disubstituted Fos (1-(3,4-Dichlorophenyl)-3-(N-hydroxyphenylamido) propylphosphonic acid) was shown to have equivalent activity (IC_50_ = 0.04 μM) as Fos. Recently, a Fos derivative (phosphonohydroxamates) was observed to inhibit parasite growth in the *in-vitro* culture of *P. falciparum* Dd2 strain, resistant to chloroquine and mefloquine (Faísca Phillips et al., 2015). In search of better derivatives of Fos, reverse analogs of Fos were also synthesized. Behrendt et al. (2011) synthesized the reverse hydroxamate based inhibitors amongst which [1-(3,4-Difluorophenyl)-4-(hydroxylamino)-4-oxobutyl] phosphonic acid is considered most effective with an IC_50_ of 3 nM. Later Brücher et al. (2012) synthesized α-Aryl-substituted β-oxa isosteres of Fos with a reverse orientation of the hydroxamic acid group and tested them against recombinant *P. falciparum* IspC and chloroquine-sensitive and resistant strains of *P. falciparum*. They found an inhibitory activity of these derivatives against *P. falciparum* IspC, with the most active derivative ((3,4-Difluorophenyl) (2-(hydroxy(methyl)amino)-2-oxoethoxy) methyl) phosphonic acid showing an IC_50_ value of 12 nM and potent *in-vitro* anti-plasmodial activity. Following similar approach for synthesis of reverse analogs, Konzuch et al. (2014) showed a new compound 4-[Hydroxy(methyl)amino]-1-(4-methoxyphenyl)-4-oxobutylphosphonic acid, to exhibits more than one order of magnitude of activity in comparison to Fos.

Another approach has been tried by Haymond et al. (2014), where several compounds from resolved crystal structures of *M. tuberculosis* MEP synthase in complex with Fos were designed, containing an amide-linked or O-linked functional group. The strategy was to target two major binding sites in MEP synthase; the Fos/DXP site and the NADPH site, bridging these adjacent sites to yield a highly specific inhibitor ligand. Amongst the compounds tested, the most effective inhibitor (diethyl 3-(benzyloxyamino) propylphosphonate) binds to both the NADPH and DXP sites, acting as a potent tight binding inhibitor of the enzyme in both *M. tuberculosis* and *Y. pestis*. However, a growth inhibition secondary screen revealed that the whole-cell inhibitory activity of this compound is relatively poor, indicating the need for additional structure-activity relationship studies to elucidate the underlying etiology.

Apart from the synthesis of Fos derivatives, various other trials are underway to increase the efficacy of Fos. Nair et al. (2011) had shown the increase in permeability and uptake of Fos in *T. gondii* in presence of recombinant GA3P transporter (GlpT) protein of *E. coli*. Similarly, Sparr et al. (2013) has recently reported a mechanism of tagging Fos with cell penetrating peptides consisting of octa-arginine which could ultimately block the hepatic stages of the parasite.

#### IspD [2-C-methyl-D-erythritol 4-phosphate cytidylyltransferase (YgbP)]

IspD (EC 2.7.7.60) enzyme, catalyzes the cytidylation process and participates in the third step of the pathway where its activity is highly dependent upon divalent cations Mg^2+^ or Mn^2+^ (Richard et al., 2001). Nucleotide derivatives, i.e., Cytosine-5′-triphosphate (CTP) and phosphate groups i.e., 2-C-methyl-D-erythritol-4-phosphate (MEP) are introduced directly as substrates in the IspD catalyzed reaction and produces 4-diphosphocytidyl-2C-methyl-D-erythritol (CDP-ME) with pyrophosphate as a bi-product. The catalytic mechanism of IspD enzyme is unique and comes under the category of associative mechanism. According to this process, when there is a nucleophile attack on the α-phosphate of CTP by the 4-phosphate of MEP, a negatively charged penta-coordinate transition state is formed. The collapse of this charged transition state finally leads to pyrophosphate release and CDP-ME formation. Two lysine residues (Lys27 and Lys213 in *E. coli* IspD) are critical to stabilize this pentavalent transition state (Richard et al., 2004). Amino acid alignments show that the basic residues in the IspD active site, in particular the *E. coli* IspD Lys27/Lys213 are also conserved in *P. falciparum* (Hunter, 2011). The X-ray crystal structure of IspD protein is known from several organisms, such as *M. tuberculosis* (PDB Id: 2WXN; Björkelid et al., 2011), *Thermotoga maritima* (PDB Id: 1VPA; unpublished), *Neisseria gonorrhoeae* (PDB Id: 1VGZ; Badger et al., 2005), *Thermus thermophilus* (PDB Id: 2PX7; unpublished), *Listeria monocytogenes* (PDB Id: 3F1C; unpublished) and *Arabidopsis thaliana* (PDB Id: 1W77; Gabrielsen et al., 2006). The presence of a conserved CDP-ME synthase domain (GT-A superfamily) is important for the functionality of IspD enzyme and follows a Rossmann fold arrangement which only allows the sequestration of pyrimidine nucleotide i.e., CTP. The presence of two signature motifs GXG and [IVT] -[LIVMC] -[IVT] -[HS] -D-[SGAV] -[AV] -R is another important feature of IspD enzyme where the Glycine rich GXG motif at N- terminal provides a proper orientation for the binding of substrate near to the other conserved signature motif of the enzyme (Shi et al., 2007).

**Figure 2:**
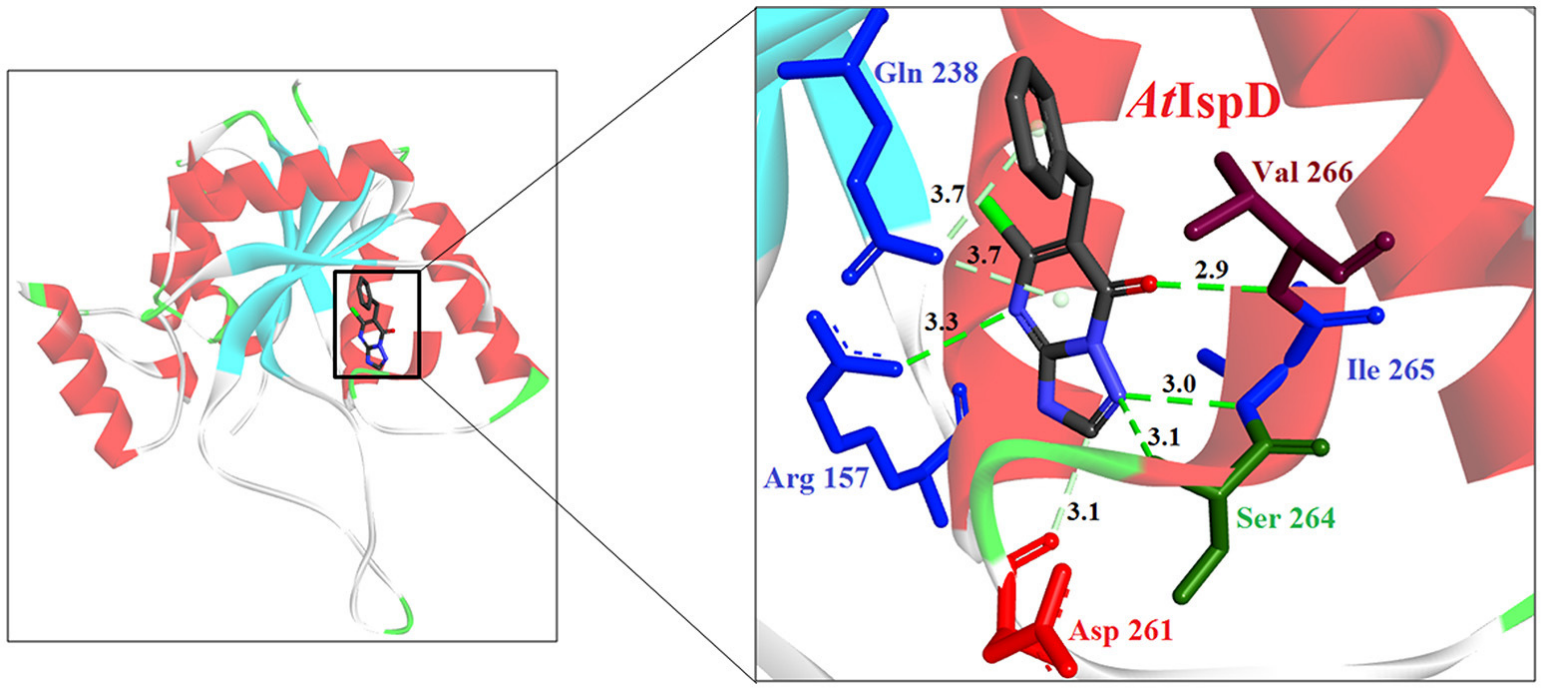
**X-ray crystal strucuture (PDB Id: 2YC3; Witschel et al., 2011) of *A. thaliana* IspD interacting with Azolopyrimidines (represented as stick)**. The inhibitory molecule 7-hydroxy-[1,2,4] triazolo [1,5-a] pyrimidine interact with R157, Q238, D261, S264, I265, and V266 residues of enzyme. CPK color scheme followed and distance represented in Å.

The first inhibitor of IspD enzyme was reported from *Brucella abortus*, where a compound named L-erythritol-4-phosphate (E4P) showed an IC_50_ of 1.36 mM (Lillo et al., 2003). Later, Witschel et al. (2011) showed the inhibition of IspD in *A. thaliana* by a synthetic compound 7-hydroxy [1,2,4] triazolo [1,5-a] pyrimidine (Azolopyrimidine). Crystallization studies of this compound with IspD showed interactions at R157, Q238, D261, S264, I265, and V266 residues (Figure 2) suggesting an allosteric inhibition. Further, direct enzyme based assays by Reker et al. (2014) suggested Pyrrolopyrazines derivatives as potent inhibitor of *A. thaliana* IspD. They have also shown the potential of these pyrrolopyrazines for inhibiting the growth of malaria parasite in cell based assays, where, 6-Amino-7-(1*H*-benzo[*d*]imidazol-2-yl)-5-[5-(diethylamino)-1-methylbut-1-yl)-5*H*-pyrrolo [3,2-*b*] pyrazine-2,3-dicarbonitrile was shown to be effective at nano molar concentration. However, the authors have also suggested an additional mechanism of action for the compound in *Plasmodium*. In another study, highly halogenated marine alkaloid of class pseudilins isolated from the marine bacterium *Pseudomonas bromoutilis* exhibited both herbicidal and anti-malarial activity (EC_50_ of 1–12 μM in cell based assays). However, cytotoxicity of these pseudilins in mammalian cell also suggests additional molecular targets apart from IspD (Kunfermann et al., 2014). Recently, another inhibitor MMV008138 (shortlisted from Malaria Box) was found effective against *Pf* IspD enzyme showing competitive inhibition with CTP substrate (Wu et al., 2015; Yao et al., 2015), whereas, in *P. vivax*, it is effective only at a lower concentration of CTP substrate (Imlay et al., 2015). In addition, MMV008138 does not exhibit activity against liver stages of *P. yoelli* nor does it have activity against sexual stages of *P. falciparum* (Bowman et al., 2014). Thus, further investigation is required for using MMV008138 as a common inhibitor for the IspD enzyme of both *P. falciparum* and *P. vivax*.

#### IspE [4-(cytidine-5-diphospho)-2-C-methyl-D-erythritol kinase (CMK)]

The fourth step of the pathway is catalyzed by IspE (EC 2.7.1.148) enzyme, an ATP and Mg^2+^ dependent enzyme belonging to the ATP–dependent GHMP kinase super-family (Andreassi and Leyh, 2004). It catalyzes the conversion of CDP-ME to 4-diphosphocytidyl-2-C-methyl-D-erythritol-2-phosphate (CDP-ME2P) in an ATP-dependent manner. The X-Ray crystal structure of IspE has been elucidated from *E. coli, T. thermophilus* HB8 (PDB Ids: 2WW4 and 1UEK; Wada et al., 2003), *Aquifex aeolicus* (PDB Id: 2VF3; Sgraja et al., 2008) and *M. tuberculosis* (PDB Id: 3PYD; Shan et al., 2011). The IspE enzyme exists as a monomer and displays the characteristic two-domain fold of the GHMP kinase superfamily. The active site of IspE is enclosed in a deep cleft between these two domains (Rohdich et al., 2000) where three known conserved motifs are present. Motif A (Lys13 to Leu18) forms the substrate binding site, Motif B (Gly102 to Ser107) forms a glycine-rich phosphate binding loop that interacts with the ATP and Motif C (Val254 to Gly258) helps to stabilize the conformation of motifs A and B rather than interacting with ligands directly (Sgraja et al., 2008; Eoh et al., 2009; Shan et al., 2011). In addition, a small, hydrophobic pocket lies adjacent to the CDP-binding site lined by amino acids Leu14, Ile27, Tyr175, and Leu208 (Hirsch et al., 2007).

**Figure 3:**
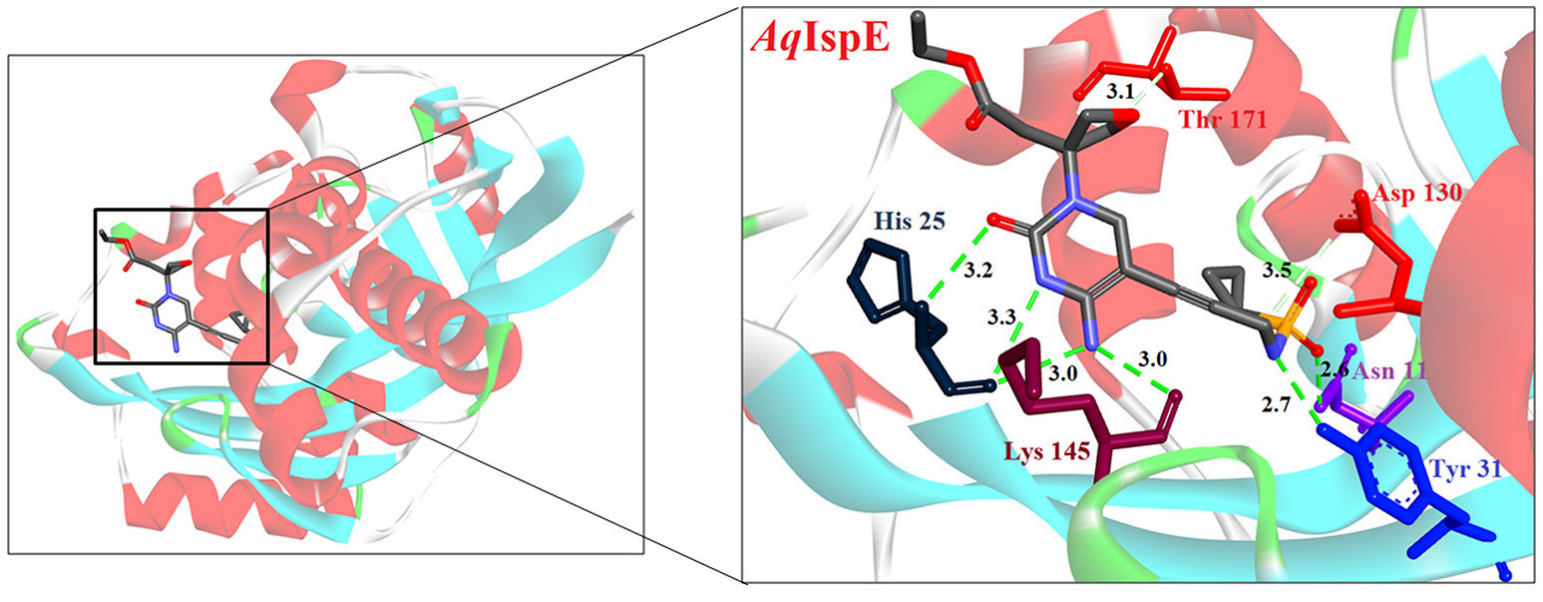
**X-ray crystal strucuture (PDB Id: 2VF3; Hirsch et al., 2008) of *A. aeolicus* IspE interacting with ethyl {3-[4-amino-5-{3-[(cyclopropylsulfonyl) amino] prop-1-yn-1-yl}-2-oxopyrimidin-1(2H)-yl] oxetan-3-yl} acetate (represented as stick)**. The inhibitory molecule interacts with N11, H25, Y31, D130, K145, and T171 residues of enzyme. CPK color scheme followed and distance represented in Å.

Hirsch et al. (2008) used the structure-based design approach and developed various inhibitors to target the substrate instead of enzyme. On screening of these compounds, Ethyl {3-[4-amino-5-{3-[(cyclopropylsulfonyl) amino] prop-1-yn-1-yl}-2-oxopyrimidin-1(2H) -yl] oxetan-3-yl} acetate was found to be the most promising candidate with an IC_50_ of 590 ± 10 nM. Further, co-crystallization studies of IspE enzyme along with this inhibitor in *E. coli* and *A. aeolicus* confirmed that the inhibitor fits properly in the cytidine-binding pocket of the enzyme, where the cyclopropyl substituent of the sulfone moiety occupies the small cavity not used by the substrate (Figure 3). The highly hydrophobic Phe185 in *E. coli* IspE is replaced with a more hydrophilic tyrosine (Tyr) residue in *M. tuberculosis, P. falciparum* and *A. aeolicus*, affecting the binding characteristics of this pocket, by strongly reducing its hydrophobic character. Thus, the cyclopropyl ring may not locate to this sub-pocket and indeed, the cyclopropyl ring prevents any solvation of the phenolic hydroxyl group of Tyr175. This explains the reduced inhibitory activity of the above compound against *A. aeolicus* and *P. falciparum* IspE (Hirsch et al., 2007, 2008).

In another study, from a list of existing small molecule inhibitors of GHMP kinases, Tang et al. (2011) identified and confirmed two novel scaffold classes of CDP-ME kinase inhibitors in *E. coli*; 6-(benzylthio)-2-(2-hydroxyphenyl)-4-oxo-3,4-dihydro-*2H*-1,3-thiazine-5-carbonitrile and (Z)-3-methyl-4-((5-phenylfuran-2-yl) methylene) isoxazol-5(*4H*)-one. Selection of these compounds was based on the predicted binding mode where 6-benzylthio and 5-phenylfuran ring moieties present in these compounds show a strong π-π interaction with cytidine binding pocket of CDP-ME created by three critical residues, viz. Tyr25, His26, and Phe185. In addition, binding of first compound show that its central core dihydro-*2H*-1,3-thiazine-5-carbonitrile –C = O mimics the α-, β-phosphates of substrate CDP-ME and participates in H-bonding interaction with Asp141 –NH…O-, whereas the 2-hydroxy-aryl ring position toward the binding site of the D-erythritol moiety of CDP-ME. IC_50_ values of these compounds were 18 and 5.5 μM, respectively. Substructure search and docking experiments based on these two scaffolds further identified 23 analogs for structure-activity relationship (SAR) studies. Three new compounds from the isoxazol-5(*4H*)-one series have shown inhibitory activities against *E. coli* and *Y. pestis* CDP-ME kinases with the IC_50_ values ranging from 7 to 13 μM (Tang et al., 2011).

Another compound which has shown inhibitory activity against *Pf* IspE is 1,3-diiminoisoindoline carbohydrazide with an IC_50_ value <100 nM in cell based assay. Synthesis of a variety of derivatives allowed an improvement of the initial antimalarial activity down to IC_50_ = 18 nM for the most potent compound (Mombelli et al., 2012). However, till date, none of the tested derivatives have shown any inhibitory activity against *P. falciparum* IspE *in-vitro* below 100 μM (Masini and Hirsch, 2014), which demands further investigation into this enzyme.

#### IspF [2C-methyl-D-erythritol-2, 4-cyclodiphosphate synthase (ygbB)]

IspF (EC 4.6.1.12) catalyzes the conversion of CDP-ME-2P into 2-*C*-methyl-D-erythritol-2,4-cyclodiphosphate (MECP), and like IspD, is dependent on divalent cations Zn^2+^ or Mn^2+^ for its activity. X-ray crystal structures of IspF from *E. coli* (PDB Id: 2GZL; Crane et al., 2006); (PDB Id: 1KNJ; Richard et al., 2002); (PDB Id: 1U3L; Steinbacher et al., 2002), *A. thaliana* (PDB Id: 2PMP; Calisto et al., 2007), *H. influenzae* (PDB Id: 1VH8; Lehmann et al., 2002), *M. smegmatis* (PDB Id: 2UZH; Buetow et al., 2007), *P. vivax* (PDB Id: 3BN6) and *P. falciparum* (PDB Id: 4C81; O'Rourke et al., 2014) have been determined, showing formation of a homo-trimeric quaternary structure. The active sites are located at the interface of two monomer units where the pocket involved in binding the phosphate moiety of the substrate is capped with a flexible loop that becomes completely ordered when the reaction product is bound. In *E. coli*, the downstream products of Isoprenoids pathway like geranyl- or farnesyl-pyrophosphate have been observed to bind IspF trimers at the central hydrophobic cavity, which suggests a possible feedback inhibition of the enzyme (Kemp et al., 2005). However, in *Plasmodium*, reports of geranyl pyrophosphate interacting with IPP in the cytoplasm (Jordão et al., 2013) indicates that though IspF is present as homo-trimer in *P. falciparum* apicoplast, it may not be affected by any feedback mechanism of geranyl pyrophosphate localized in cytoplasm.

**Figure 4:**
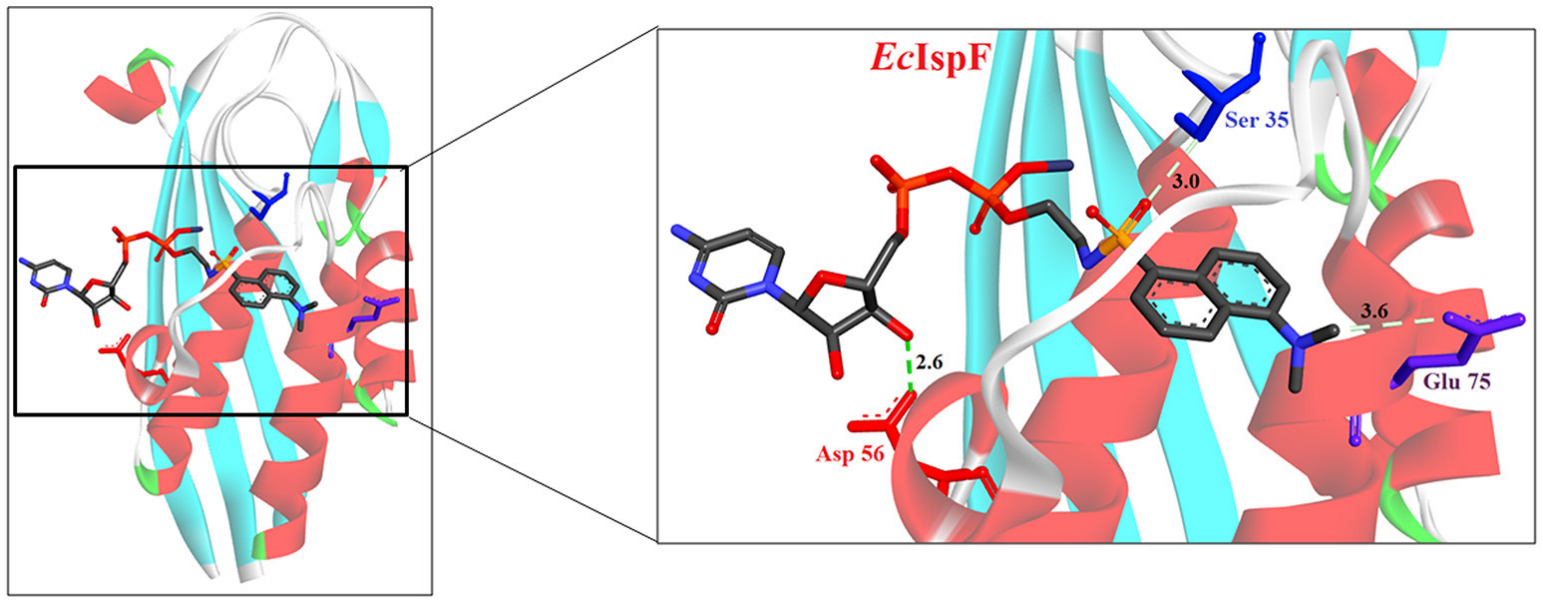
**X-ray crystal strucuture (PDB Id: 2GZL; Crane et al., 2006) of *E. coli* IspF interacting with diammonium 5′-O-{[({[2-({[5-(Dimethylamino) naphthalene-1- yl]sulfonyl}amino) ethyl] oxy}phosphinato)oxy] phosphinato} cytidine (represented as stick)**. The fluorescent inhibitory molecule interacts with S35, D56, and E77 residues of enzyme. CPK color scheme followed and distance represented in Å.

Despite the fact that the active site of IspF is considered the most druggable, based on the presence of high apolar amino acid residues, very few inhibitors have been reported. Crane et al. (2006) tested various anthranilate compounds with cytidine moiety, amongst which fluorescent diammonium 5′-O-{[({[2-({[5-(Dimethylamino) naphthalene-1- yl] sulfonyl} amino) ethyl] oxy} phosphinato) oxy] phosphinato} cytidine was found to be effective against *E. coli* IspF. In *in-vitro* studies, this inhibitor was reported to interact with S35, D56 and E77 residues of *E. coli* IspF enzyme (Figure 4). The most successful inhibitors identified till date for IspF are non-cytidine-like thiazolopyrimidine derivatives, with high activity against both *P. falciparum* (IC_50_ = 9.6 μM) and *M. tuberculosis* IspF (IC_50_ = 6.1 μM) (Geist et al., 2010), and the aryl bis-sulphonamide inhibitors showing inhibition of *P. falciparum* IspF and *A. thaliana* IspF with IC_50_ values as low as 1.4 μM and 240 nM, respectively (Thelemann et al., 2015). However, their binding mode and optimization is yet to be reported.

#### IspG [4-hydroxy-3-methyl-2-(E)-butenyl-4-diphosphate synthase (gcpE)] and IspH [4-hydroxy-3-methyl-2-(E)-butenyl-4-diphosphate reductase (lytB)]

IspG and IspH enzymes participate in the last two steps of the pathway, respectively. Initially, IspG catalyzes the reduction of MECP through a multistep reaction and converts it into 4-hydroxy-3-methyl-2-(*E*)-butenyl-4-diphosphate (HMBDP) and then IspH converts it to IPP and DMAPP (Rohdich et al., 2002). The X-ray crystal structure of IspG has been derived from *A. aeolicus* (PDB Id: 3NOY; Lee et al., 2010) and *T. thermophilus* (PDB Id: 2YOF; Rekittke et al., 2011). The IspG enzyme consists of two conserved domains: an N-terminal TIM barrel domain for the binding of MECP substrate and a C-terminal domain for binding of Fe-S clusters. In *P. falciparum*, the electrons required for the binding of MECP substrate to the IspG enzyme are provided by ferredoxin/ferredoxin NADP+ reductase system through [4Fe-4S] clusters (Röhrich et al., 2005). This binding of the MECP substrate is responsible for the formation of a double bond that converts cyclic form of the MECP molecule to aliphatic HMBDP.

The HMBDP formed by IspG is then converted to the IPP and DMAPP by IspH enzyme and this conversion consists of three steps: (i) removal of a hydroxyl group, (ii) transfer of two electrons from the [4Fe-4S] cluster, and (iii) the protonation of an intermediate allylic anion (Laupitz et al., 2004). The X-ray crystal structure of IspH has been derived from *E. coli* (PDB Id: 3KE8; Gräwert et al., 2010), *A. aeolicus* (PDB Id: 3DNF; Rekittke et al., 2008) and *P. falciparum* (PDB Id: 4N7B; Rekittke et al., 2013). IspH consists of two domains, a LytB domain for the binding of HMBDP and a Fe-S cluster binding domain similar to IspG. Specific requirements for the activity of IspG and IspH enzyme was proven by a study in *Saccharomyces cerevisiae*, where the reconstruction of the complete MEP pathway was aimed, however, even after incorporation of all the necessary components including all the enzymes, substrates and cofactors, the pathway remained non-functional. It was concluded that a reducing environment and compartmentalization is required for the functionality of these enzymes (Partow et al., 2012).

**Figure 5:**
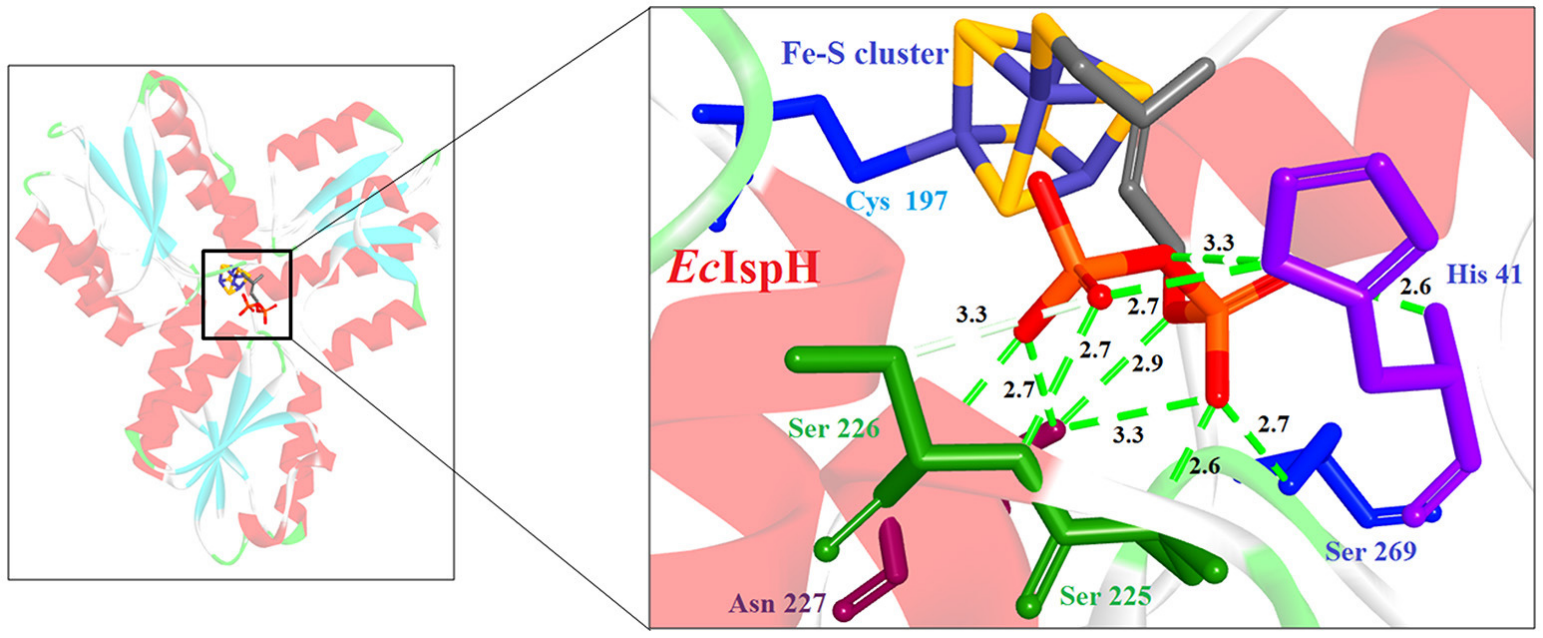
**X-ray crystal strucuture (PDB Id: 4H4E; Span et al., 2013) of *E. coli* IspH interacting with (E)-4-mercapto-3-methyl but-2 enyl diphosphate (represented as stick)**. The inhibitory molecule interacts with H41, S225, S226, N227, and S269 residues of enzyme. CPK color scheme followed and distance represented in Å.

Most of the inhibitors developed initially against IspG and IspH targeted the Fe-S clusters, especially the unique fourth iron site, but as most of the metallo-proteins in mammals, including humans require these Fe-S clusters for their activity, these inhibitors have selectivity issues. Substrate analogoues that bind to IspG and IspH have been tried, but they could not turn out to be potent inhibitors. Replacing the diphosphate group with another moiety like carbamates or aminosulfonyl carbamates showed only very weak inhibitory activity (Van Hoof et al., 2008). In search of inhibitor, amongst various chemically synthesized compounds, (E)-4-mercapto-3-methyl but-2 enyl diphosphate (Alkyne diphosphate derivative) was shown to inhibit *E. coli* IspH enzyme (Figure 5) where interaction was observed with H41, S225, S226, N227, and S269 (Xiao et al., 2011; Span et al., 2013). E126 residue is reported to be important for the activity of IspH enzyme in *E. coli* and *A. aeolicus*, and a mutation at this residue can cause a significant effect on its activity (Wang et al., 2010). This residue is conserved in other organisms, including Apicomplexans and plants.

Bhuyan et al. (2015) screened a large ligand data-set containing diphospate group against the *P. falciparum* IspH structure, and based on Goldscore and Chemscore identified 17 lead compounds amongst which 5-((hydroxymethyl)-O-pyrophosphoryl) uracil shows best binding affinities with *Plasmodium* IspH, and thus can be considered as its potential inhibitor. Recently, the derivatives of diphosphonate such as alkyl phosphate have been identified as potential inhibitors for IspG and IspH enzymes of *A. aeolicus, E. coli*, and *P. falciparum* (Guerra et al., 2014), however their exact mechanism for inhibition is unknown.

### MEP pathway as a potential drug target in *Plasmodium*

MEP pathway and its enzymes present attractive new targets for the development of broad-spectrum novel anti-microbial and anti-malarial drugs. In the last decade, various researchers have extensively explored these enzymes and their active sites to identify/design probable inhibitors in prokaryotes (Hale et al., 2012). *In-silico* studies and certain *in-vitro* assays performed on the malaria parasite culture using drugs tested on prokaryotic homologs have detailed few compounds that can block the parasite growth by specifically binding to the enzymes of MEP pathway. These compounds can be further analyzed for their pharmacokinetics and pharmacological efficacy to establish them as probable anti-malarials.

The natural antibiotic Fos, an inhibitor of IspC/DXR enzyme has been widely used as an anti-bacterial and as an anti-malarial to block *in-vitro* parasite cultures. Despite the apparent merits of Fos, the drawbacks associated with it like low absorption and shorter half-life, hampered its market introduction. Several research groups attempted to improve its structural activity by chemical modification, but none has been able to achieve activity similar to Fos *in vivo*. However, the natural acetyl derivative of Fos, FR900098, when tested for its anti-malarial property has shown better activity (IC_50_ = 0.018 μM) than Fos. Thus, an extensive research could help in the development of new chemically synthesized derivatives of Fos and similar compounds, to be used as potential anti-malarial.

With the studies performed on the initial enzymes of the *Plasmodium* MEP pathway, showing the essentiality and the uniqueness of the pathway for the survival of the parasite, it becomes imperative to study the remaining enzymes of the pathway. With the availability of the X-ray crystal structure of almost half of them (IspC/DXR, IspF & IspH) from *P. falciparum*, showing the conserved functional domains and substrate binding sites, extrapolating the prokaryotic MEP inhibitors data to *Plasmodium* is a promising way of identifying novel drugs. Another approach can be to test the efficacy of clinically approved and commercially established anti-bacterials targeting these enzymes, as initially Fosmidomycin was also one amongst those compounds.

## Author contributions

GS, SG, and VS conceptualized and designed the manuscript. GS and VS drafted the manuscript. GS, ZP, SG, and VS read and critically revised the contents of the manuscript.

### Conflict of interest statement

The authors declare that the research was conducted in the absence of any commercial or financial relationships that could be construed as a potential conflict of interest.
