# Supplementary material for: New Insight into Isoprenoids Biosynthesis Process and Future Prospects for Drug Designing in Plasmodium
Source: Front Microbiol. 2016 Sep 13;7:1421. doi: 10.3389/fmicb.2016.01421 (PMC5020098; doi:10.3389/fmicb.2016.01421)
Supplement: Supplementary file 1 [file DataSheet1.DOCX]

**Supplementary Data**

| **Figure S1: Isoprenoid biosynthesis by MVA and MEP pathways:** Isoprenoids are synthesized from 5-carbon intermediate Isopentenyl pyrophosphate (IPP) and its isomer, Dimethylallyl pyrophosphate (DMAPP). Synthesis of IPP and DMAPP can take place by two pathways – MVA & MEP. The MVA pathway (S1a) initiates with the condensation of acetyl CoA. Enzymes participating in this pathway are ACT, HMGS, HMGR, MVK, PMK and MDS. In the MEP/ DOXP pathway (S1b), IPP and DMAPP are generated from pyruvate and glyceraldehydes-3-phosphate. Enzymes of this pathway are named here according to their *E. coli* homologues (DXS, IspC, IspD, IspE, IspF, IspG and IspH). | |
| --- | --- |
| 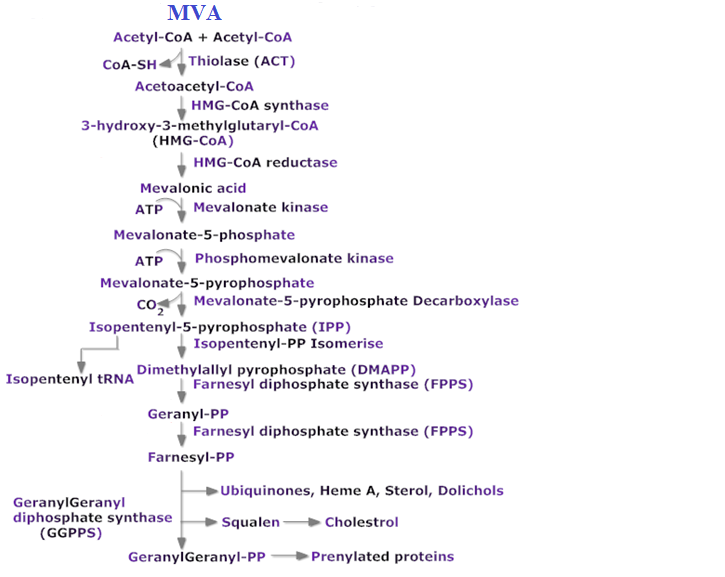 | 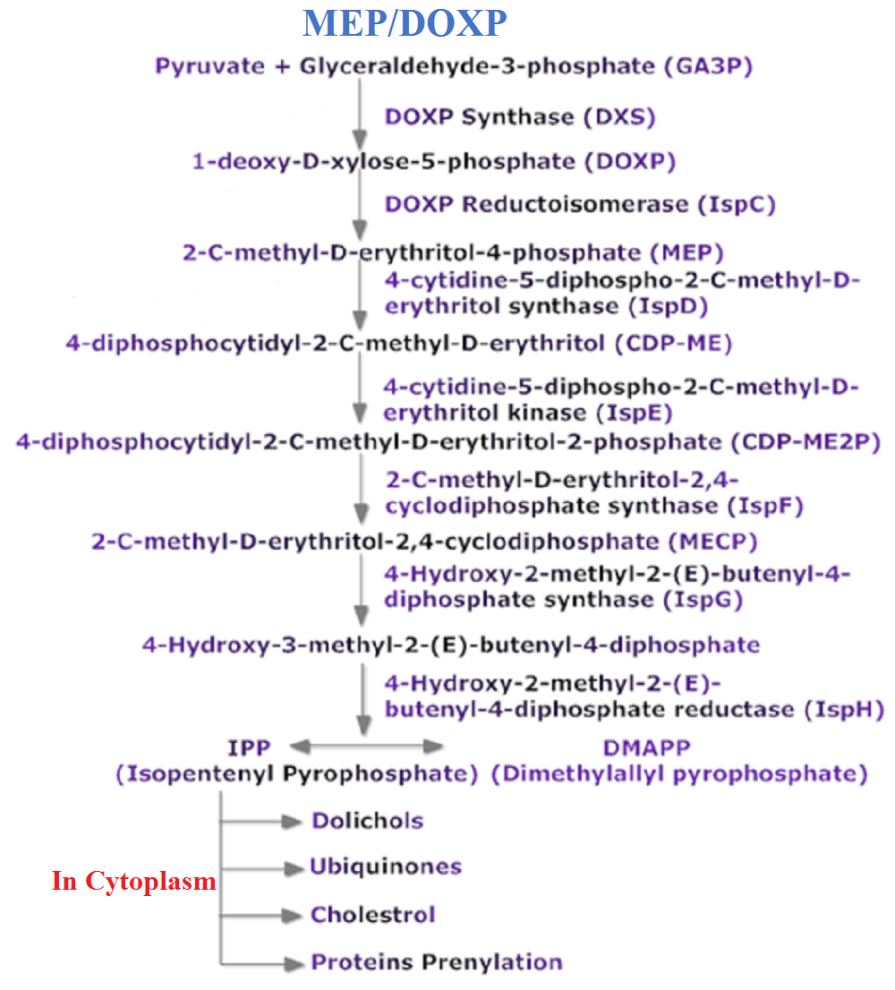 |
